# Exploring the Role of Carbon Monoxide in Seed Physiology: Implications for Stress Tolerance and Practical Uses

**DOI:** 10.3390/ijms26010223

**Published:** 2024-12-30

**Authors:** Faezeh Bazvand, Łukasz Wojtyla, Hamid Reza Eisvand, Małgorzata Garnczarska, Małgorzata Adamiec

**Affiliations:** 1 Department of Plant Production Engineering and Genetics, Faculty of Agriculture, Lorestan University, Khorramabad 68151-44316, Iran; f.bazvand95@gmail.com (F.B.); eisvand.hr@lu.ac.ir (H.R.E.); 2 Department of Plant Physiology, Institute of Experimental Biology, Faculty of Biology, Adam Mickiewicz University, Poznań, ul. Uniwersytetu Poznańskiego 6, 61-614 Poznań, Poland; wojtylal@amu.edu.pl (Ł.W.); msolin@amu.edu.pl (M.A.)

**Keywords:** antioxidant defense, carbon monoxide, germination, photosynthesis, root development, seed biology, stress response

## Abstract

Carbon monoxide (CO) is recognized as a signaling molecule in plants, inducing various physiological responses. This article briefly examines the physiological functions of CO in seed biology and seedlings’ responses to environmental stresses. The activity of heme oxygenase (HO), the main enzyme responsible for CO synthesis, is a key factor controlling CO levels in plant cells. CO can influence seed germination by regulating seed dormancy through interactions with genes and hormones. Additionally, CO positively affects seedling growth by enhancing the antioxidant system, thereby increasing resistance to oxidative damage caused by stress. CO has beneficial effects on root development, root length, stomatal closure, and regulation of the photosynthetic system. Its interaction with reactive oxygen species (ROS) mediates hormone- and light-dependent growth processes during the early stages of plant development under stress. Furthermore, CO interacts with other signaling molecules, such as nitric oxide (NO), molecular hydrogen (H_2_), and hydrogen sulfide (H_2_S). By gaining a better understanding of the molecular mechanisms underlying these processes, CO can be more effectively utilized to improve seed germination and seedling growth in agricultural practices.

## 1. Introduction

Gasotransmitters are endogenous gas molecules that play a crucial role in cellular signaling. These gases include nitric oxide (NO), carbon monoxide (CO), hydrogen (H_2_), hydrogen sulfide (H_2_S), sulfur dioxide (SO_2_), and methane (CH_4_) [1]. Approximately 10% of carbon in the universe exists as CO [2]. CO, also known as the “silent killer” is a stable, low-molecular-weight gas that plays a significant role in signal transmission [1]. Scientific interest in carbon monoxide (CO) began in the 17th century, with detailed chemical and toxicological studies following in the 18th. Despite its dangerous reputation, some scientists in the 18th and 19th centuries proposed therapeutic uses for CO. Since 2000, CO has been re-evaluated and is now seen as a neurotransmitter and a potential pharmaceutical agent [2]. Initial research onto CO’s effect on plant physiology began in the early 20th century when Richards and MacDougal [3] observed that CO toxicity disrupted plant growth and chlorophyll production in species like *Vicia faba*, *Zea mays*, and *Sinapis alba*. They found that CO is highly toxic when it replaces atmospheric nitrogen, negatively affecting plant growth, secondary membrane formation, and chlorophyll production. Although primarily focused on toxicity, their work laid the foundation for understanding plants’ responses to gaseous environmental changes. Currently, we know that in plants, CO functions as a signaling molecule, influencing vital physiological processes such as seed dormancy and germination, root development, and stomatal closure [1,4]. It also enhances plant resistance to abiotic stresses, including drought, salinity, and heavy metal exposure, by inducing the production of defense-related hormones. CO also contributes to intracellular and intercellular functions, including antioxidant enzyme expression and programmed cell death (PCD) modulation [5,6]. Furthermore, CO interacts with other signaling molecules, such as phytohormones and hydrogen peroxide (H_2_O_2_), to regulate plant growth and stress responses [5,7,8].

This review summarizes the molecular mechanisms by which CO regulates seed physiology and supports resilience under abiotic stress conditions.

## 2. Formation of Endogenous CO

**Figure 1:**
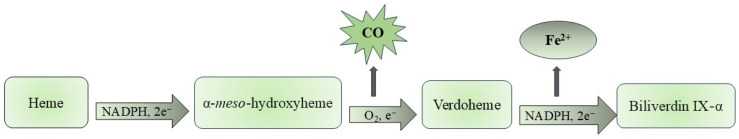
CO synthesis through the degradation of heme by heme oxygenase.

CO is not only exogenous to plants but also produced intracellularly during heme degradation, primarily through the activity of heme oxygenases (HO) [9]. These enzymes, in the presence of molecular oxygen and electrons supplied by NADPH [10], break down heme to form biliverdin IXα (BV) and release iron (Fe^2+^). The biliverdin is subsequently converted into bilirubin by biliverdin reductase (Figure 1).

In *Arabidopsis thaliana*, four nuclear-coded heme oxygenases (HY1, HO2, HO3, and HO4) with chloroplast transit peptides at their N-terminus have been identified [11]. These proteins are divided into the HO1 and HO2 subfamilies. The HO1 subfamily includes HY1, HO3, and HO4, while HO2 is the sole member of the HO2 subfamily. Both subfamilies were also found in other plants like soybean, tomato, sorghum, rice, and pine [11]. The main difference between these subfamilies is a spacer sequence in the HO2 subfamily, rich in glutamate, aspartate, and glycine residues. Additionally, members of the HO2 family lack a conserved histidine residue in the active site, which is essential for heme iron coordination, and therefore have no ability to cleave the heme bond [12].

CO exhibits its biological activity by forming bonds with metals in the active centers of proteins, particularly those containing the heme group [10]. However, the specific chemical interactions of CO with heme-containing proteins in plant cells have not yet been confirmed. CO production in plants increases under stress conditions, and its role in stress response and its significant impact on dormancy release and seed germination is undeniable but poorly understood. Knowledge about these processes could benefit agrotechnical strategies to enhance plant productivity.

## 3. The Role of CO in Root Development

Roots are essential for plant survival, facilitating nutrient and water uptake. Their development involves cell communication that regulates meristematic differentiation [13]. Recent studies have highlighted the significant role of CO in enhancing root development during the early seedling stage. The observed effects of CO treatment on root development in the species studied are summarized in Table 1. CO has been shown to be an important regulator of root formation in plants. In *Solanum lycopersicum*, exogenous application of CO increased the number of lateral roots and primary root elongation [14] as well as the density and length of root hairs [15]. The observed effects of CO application were dependent on its concentration and related to heme oxygenase-1 activity, since tomato mutants deficient in this enzyme delayed root hair development. Similarly, application of hematin, which acts as a CO donor and inducer of HO, effectively promoted root formation in *Phaseolus radiatus* hypocotyl cuttings rooting. Also, the observed effects were dose- and time-dependent [16].

In addition, CO also plays an important role in enhancing root development in response to various stresses. Under salt stress, application of exogenous hematin significantly increased root length in *Sorghum bicolor* [17] and *Oryza sativa* [18]. Also, application of exogenous CO to young seedlings of *Triticum aestivum* exposed to salt stress resulted in increased root dry weight and relative water content (RWC) [19]. Under drought conditions, hemin treatment [20] has been shown to stimulate seedling growth and promote adventitious root growth in *Solanum lycopersicum* [21] and *Cucumis sativus* [22]. In addition, increased RWC was observed in *Cucumis sativus* roots, suggesting that CO helps to maintain water balance [22]. The importance of CO in response to stress was further emphasized by the fact that it also plays an important role in response to oxidative stress, which is one of the early responses in plants to various abiotic and biotic stresses. CO was found to play a protective role in *Medicago sativa* roots exposed to cadmium (Cd) stress by alleviating oxidative damage [7]. Increased HO-1 activity leads to higher CO levels, helps alleviate Cd-induced oxidative damage, and reduces Cd accumulation in plant tissues. Application of CO donors such as hemin promotes the expression of antioxidant genes such as *Mn-SOD* and *POD*, thereby enhancing the plant’s antioxidant defense system [23].

The molecular mechanism of CO action appears to be closely related to antioxidant mechanisms. A study of wheat seedlings under salt stress showed that the application of a low concentration of a CO donor reduced lipid peroxidation and increased the activities of key antioxidant enzymes such as guaiacol peroxidase (POD), superoxide dismutase (SOD), ascorbate peroxidase (APX), and catalase (CAT). These protective effects were explicitly confirmed by CO, as hemoglobin (CO scavenger) blocked the observed effects [24]. CO release and increase in HO activity were also found to help delay programmed cell death (PCD) by reducing the overproduction of superoxide anion and the activity of NADPH oxidase, while increasing the activity of superoxide dismutase (SOD) and the expression of its isoforms (Mn-SOD and Cu/Zn-SOD) [25].

Yun et al. [26] identified 1792 genes whose expression was significantly altered in CO-treated seedlings compared to control seedlings. These genes included a group involved in important metabolic pathways such as plant hormone signaling, sucrose, starch, and phenylalanine metabolism. Closer examination revealed some specific genes involved in the rooting process. Increasing the expression of these genes increased the plant’s accumulation of hormones such as auxin, jasmonic acid (JA), and salicylic acid (SA). It also increased the amount of starch and total sugars in the plant. Some other genes were also involved in the structure of the cell wall, which is necessary for the formation of new roots [26]. Despite this advances in understanding the role of CO in plant rooting, several gaps remain, including the molecular mechanisms, concentration-dependent effects, environmental interactions, crosstalk with other signaling gases, species-specific variability, and the long-term effects on root architecture and plant productivity. These unresolved areas highlight the need for further investigation into the complex signaling and physiological roles of CO.

**Table 1:** Overview of CO impact on physiological processes in seed and seedling.

Plant Species	CO-Concentration	CO Application Stage	Stress Type	CO-Induced Effect	Reference
*Arabidopsis thaliana*	CORM-2 (1 µM)	Germination	_	Increased germination percentage; decreased seed dormancy	[4]
*Arabidopsis thaliana*	40 µM COMR-2	Germination	_	Increased stomatal index (SI)	[27]
*Arabidopsis thaliana*	5 μM CORM-2	Prior to germination	_	Increased germination rate, amylase activity	[28]
*Arabidopsis thaliana*	Exogenous CO 50 µM (15–20 min)	Seedling	Fe-EDTA (50 µM)	Improve chlorophyll content; up-regulated expression of *AtFIT1*, *AtFRO2*, *AtFER1*, and *AtIRT1*	[29]
*Baccaurea ramiflora*	10 mM hematin, 50% CO-saturated solution (15–20 min)	Germination	Chilling (2 °C, 2 h)	Increasing germination rate, the activity of APX, GR, monodehydroascorbate reductase (MDHAR), and S-nitrosoglutathione reductase (GSNOR); decreased content of H_2_O_2_, and RNS	[30]
*Brassica juncea*	0.2 mM CO	Seedling	Hg stress (10 mM)	Enhanced root elongation, POD, APX, proline; decreased TBARSs	[22]
*Cassia obtusifolia*	5% CO-saturated aqueous solution (50 min), 1.0 μM hematin	Germination	Salinity (100 mM NaCl)	Increased germination vigor, germination rate, germination index, vigor index, radicle length, plumule length, fresh weight, chlorophyll a, chlorophyll b, total chlorophyll; decreased TBARSs, LOX activity, H_2_O_2_	[31]
*Cucumis Sativus*	30% CO aqueous solution, 500 mM Hemin	Seedling	Drought (polyethylene glycol 6000) 0.3%	Increased total chlorophyll and chlorophyll fluorescence parameters Fv/Fm, ΦPSII, and enhanced activity of CAT, POD, SOD and APX; decreased TBARSs	[32]
*Cucumis sativus*	10% CO aqueous solution, hematin and hemin (10 µM)	Seedling	ZnPPIX (200 mM)	Enhanced root number explant, root length	[33]
*Medicago sativa*	50% CO saturated aqueous solution, 50 µM hemin	Seedling	Paraquat (5 µM)	Increased root elongation, POD, SOD, and APX; decreased TBARSs	[23]
*Nicotiana tabacum*	500 mM Hemin, 30% (*w*/*v*) CO aqueous solution	Seedling	Drought (polyethylene glycol 6000)	Increased development of adventitious roots, RWC, chlorophyll a, chlorophyll b, fluorescence parameters; decreased TBARSs, H_2_O_2_	[34]
*Oryza sativa*	5% CO-saturated aqueous solution (15 min), 1.0 mM hematin	Germination	Salinity (100 mM NaCl)	Increased germination rate, germination energy, germination index, root length, shoot length, amylase, CAT, SOD, total soluble sugar and reducing sugar	[18]
*Phaseolus radiatus*	Hematin (10 µmol/L), 100% CO-saturated aqueous solution (15 min)	Germination	_	Increased root number explant, root number length	[16]
*Setaria faberii*	Gas atmosphere 1% CO	Germination	_	Increased germination percentage	[35]
*Solanum lycopersicum*	CO-saturated aqueous solution 10 µM (15–20 min)	Seedling	_	Increased LR number seedling, PR elongation, auxin concentration of root, stem and leaf	[14]
*Solanum lycopersicum*	CO-saturated aqueous solution 10 µM (15–20 min)	Seedling	_	Enhanced density of RH number, and length of RHs	[15]
*Solanum lycopersicum*	Hemin (500 µM)	Seedling	Drought (polyethylene glycol 6000) 5%	Increased root length, plant height, chlorophyll *a*, chlorophyll *b*	[21]
*Sorghum bicolor*	1 µM Hematin	Germination	Salinity (NaCl 250 mM)	Increase germination index, root length, and proline; decrease Na+/K+ ratio, H_2_O_2_, and metaxylem pit size	[17]
*Triticum aestivum*	1.0 mM hematin	Germination	Drought (polyethylene Glycol-6000) 25%	Increased germination percentage, amylase activity, SOD, APX, CAT, DHA reductase (DHAR), and reducing sugar content; decreased TBARSs	[36]
*Triticum aestivum*	1.0% CO aqueous solution (15 min), 1.0 µmol/L hematin	Germination	Salinity (250 mmol/L NaCl)	Increased germination rate, germination energy, germination index, CAT, APX, GPOX, SOD, Soluble sugar, reducing sugar; decreased TBARSs	[16]
*Triticum aestivum*	0.01 µmol/L hematin	Seedling	Salinity (300 mmol/L NaCl)	Increased dry weight, and RWC in shoots and roots, and activity of SOD, APX, CAT, POD; decreased TBARS content	[24]
*Triticum aestivum*	50% CO aqueous solution (30 min)	Seedling	Salinity (NaCl 400 mM)	Increased root growth, SOD; decreased NADPH oxidase	[25]
*Triticum aestivum*	1.0% CO-saturated aqueous solution (30 min), 10 μM hematin	Seedling	Light deficiency	Increased chlorophyll content, relative fluorescence, relative HO-1 expression	[37]
*Triticum aestivum*	hemin (0.1, 0.01 µM)	Seedling	Paraquat (10 mg/L), H_2_O_2_ (150 mmol/L)	Increased chlorophyll content, APX, SOD, CAT; decreased TBARSs	[38]
*Triticum aestivum*	50% CO aqueous solution (15 min)	Seedling	Salinity (NaCl 150 mM)	Increased root dry weight, shoot dry weight, root RWC, shoot RWC, APX, GR, SOD, K/Na ratio; decreased TBARSs	[19]
*Vicia faba*	Hematin (1.0 µM), 10% CO aqueous solution (30 min)	Seedling	_	Decreased stomatal aperture	[39]
*Vicia faba*	400 mM hematin, 100% CO-saturated solution (15 min)	Seedling	_	Decreased stomatal aperture	[40]

## 4. Stomata Movements and Photosynthesis

CO is one of the signaling molecules that exert a significant influence on both stomatal movement and development. These structures, located on the epidermis of cotyledons, leaves, and stems, facilitate gas exchange and respond rapidly to various environmental stimuli. The stomatal aperture plays a crucial role in optimizing the processes of photosynthesis and transpiration [27]. As demonstrated in Table 1, the treatment of *Arabidopsis thaliana* seeds with the CO-releasing molecule tricarbonyl dichloro ruthenium (II) dimer (CORM-2) results in an augmentation of the stomata index, defined as the ratio of stomata to the total number of stomata and epidermal cells. Furthermore, it has been demonstrated that both hematin and CO treatment induce stomatal closure in *Vicia faba* seedlings [39,40], and this effect appears to be independent of both dose and exposure time.

The impact of CO on photosynthetic efficiency extends beyond restricting gas exchange; it also influences the functional state of photosystem II. Research has demonstrated that the CO-saturated aqueous solution and hematin protect photosystem II (PSII) from salt stress damage. In *Cassia obtusifolia* L., these treatments have been shown to reduce the non-photochemical quenching coefficient (NPQ), suggesting a decrease in the activity of mechanisms responsible for dissipating excess light energy. Conversely, the photochemical efficiency of photosystem II (qP) and the actual PSII photochemical efficiency (*Φ*PSII), which both refer to the effectiveness of converting absorbed light into chemical energy during photosynthesis, exhibited an increase [31].

Elevated levels of CO have been found to confer protective effects on chlorophyll levels under stress conditions. In a study on drought-stressed *Solanum lycopersicum*, it was observed that treatment with hemin led to an increase in chlorophyll *a* and chlorophyll *b* levels [21]. A similar effect was observed in drought-exposed *Cucumis sativus* seedlings. The treatment of *Cucumis sativus* seedlings with both CO and hemin resulted in elevated total chlorophyll levels, which translated into enhanced chlorophyll fluorescence parameters (Fv/Fm, ΦPSII). This was accompanied by elevated activity of CAT, POD, SOD, and APX, along with a reduction in TBARS levels [22]. In *Triticum aestivum* seedlings, the application of CO and hematin resulted in augmented chlorophyll content under conditions of low illumination [37].

The protective role of CO on chlorophyll content is attributed to its role in the regulation of the chlorophyll biosynthesis pathway. In *Solanum lycopersicum*, CO treatment has been observed to increase the levels of intermediate products in the chlorophyll biosynthesis pathway, including protoporphyrin IX (Proto IX), Mg-protoporphyrin IX (Mg-Proto IX), protochlorophyllide (Pchlide), and heme, while concurrently decreasing uroporphyrinogen III (Uro III) content. Furthermore, CO has been observed to up-regulate the expression of genes associated with chlorophyll and heme synthesis (*SlPPOX*, *SlFECH*, *SlMGMT*, *SlUROD*, *SlChlS*, *SlPOR*), thereby mitigating the deleterious effects of drought stress on tomato growth. The beneficial effects of CO are reduced by hemoglobin, a CO scavenger, indicating the specific role of CO in these processes [21].

The role of CO in the regulation of photosynthesis is still not fully understood. The most intriguing questions concern its molecular impact and crosstalk with other signaling molecules, such as ROS and phytohormones. Additionally, its direct impact on Rubisco activity and role in carbon fixation should be studied in detail, especially in the context of plant productivity. Moreover, potential applications in agricultural practices to improve crop productivity and stress resilience should be thoroughly investigated.

## 5. Germination

Germination is a critical and distinct stage in a plant’s life cycle, which is essential for successfully establishing seedlings and significantly impacting future development, growth, and productivity. This stage is subject to regulation by various environmental and endogenous factors [41]. One potential mechanism by which CO may regulate seed germination involves the role of this gas in overcoming seed dormancy. Seed dormancy, defined as the state that hinders the germination of seeds under suboptimal conditions, has the potential to adversely impact the quality and yield of crops by affecting agronomic traits [42].

A primary protein that plays a regulatory role in seed dormancy is the Delay of Germination 1 (DOG1) protein [43]. Elevated levels of DOG1 have been observed to be associated with seed dormancy. Treatment of seeds with a CO donor has been shown to reduce the level of DOG1. CO functions as a signal that activates the ethylene-responsive transcription factor 12 (ERF12), a gene regulatory protein that controls the expression of specific genes. The interaction of ERF12 with the DOG1 promoter results in the repression of the *DOG1* gene expression. Consequently, CO has been shown to break seed dormancy by activating ERF12 and reducing *DOG1* gene expression. Additionally, CO contributes to this process by reducing histone acetylation in the chromatin region of the *DOG1* gene [4]. It has been demonstrated that another CO-releasing molecule, a tricarbonyl dichloro ruthenium (II) dimer (CORM-2), stimulates germination in *Arabidopsis thaliana* [4,28].

In contrast, the zinc-finger protein SOMNUS (SOM) functions as a negative regulator of germination. SOM exerts its influence on signaling pathways associated with plant hormones, including gibberellic acid (GA) and abscisic acid (ABA). GA has been shown to promote germination, while ABA has been demonstrated to inhibit this process. The presence of SOM has been shown to reduce sensitivity to GA and increase sensitivity to ABA, thereby preventing rapid germination under conditions that are not conductive to seedling development [44]. The inhibitory effect of SOM on germination is significantly reduced by CO. This inhibition decreases the plant’s sensitivity to ABA and increases its sensitivity to GA, promoting germination. Furthermore, CO plays a role in attracting histone deacetylase proteins to the *SOM* gene promoter region, thereby reducing histone acetylation. This reduction in acetylation serves to close the chromatin structure, thereby decreasing *SOM* gene expression and further enhancing germination under suitable light conditions [28].

As indicated in Table 1, CO has also been demonstrated to promote germination under abiotic stress conditions. Treatment with an aqueous solution saturated with CO and hematin has been observed to positively affect the germination of *Oryza sativa* L. seeds under salt stress by increasing amylase activity and the accumulation of soluble and reducing sugars [18]. A similar outcome was reported by Xu et al. [16] in *Triticum aestivum* L., who found that treatment with CO and hematin enhanced germination rate, energy, and index under salt stress. This enhancement was achieved by increasing the activity of catalase (CAT), ascorbate peroxidase (APX), glutathione peroxidase (GPOX), and superoxide dismutase (SOD), along with higher accumulation of soluble and reducing sugars, and a decreased level of thiobarbituric acid reactive substances (TBARSs). In another study on *Triticum aestivum* L. seedlings treated with CO, Xie et al. [19] observed increased shoot dry weight, shoot RWC, K^+^/Na^+^ ratio, activity of APX, glutathione reductase (GR), and SOD and reduced TBARS accumulation under salt stress. In a separate study, Zheng et al. [31] observed that under salt stress, treatment of *Cassia obtusifolia* L. seeds with CO or hematin increased germination vigor, germination energy, and germination index. Additionally, plumule length and fresh weight were stimulated with decreased levels of TBARSs and H*_2_*O*_2_* and lower lipoxygenase activity (LOX). Similarly, in *Sorghum bicolor* L. seeds, Ikebudu et al. [18] showed that treatment with hematin under salt stress improved the germination index, increased proline content, and decreased H_2_O_2_ and Na^+^/K^+^ ratio. Further evidence for overlap of CO and H_2_O_2_ signaling pathways was found during research concerning seed osmopriming. Specifically, it was demonstrated that heme oxygenase activity exhibited an increase in alfalfa seeds under H*_2_*O*_2_* treatment, thereby enhancing seed germination [45].

**Figure 2:**
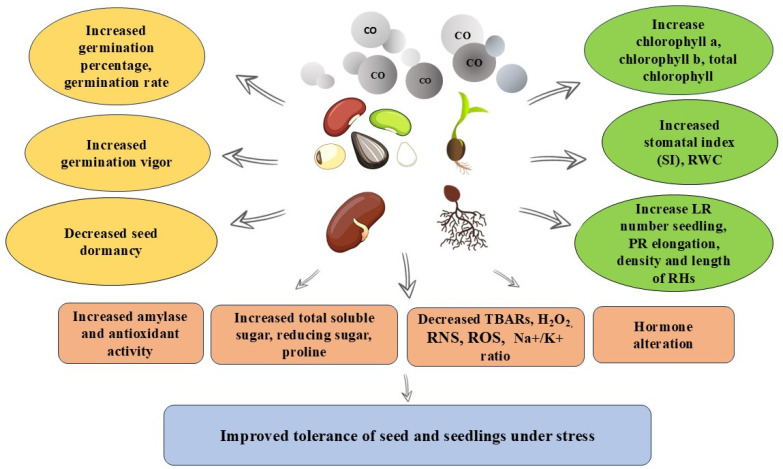
Overview of physiological and biochemical responses of seeds and seedlings to CO treatment.

The exogenous application of CO and treatment with hematin also increased the tolerance of *Baccaurea ramiflora* seeds to chilling stress [30]. This treatment resulted in higher accumulation of glutathione (GSH), enhanced activity of antioxidant enzymes involved in the glutathione-ascorbate cycle, and reduced levels of H_2_O_2_, reactive nitrogen species (RNS), and reactive oxygen species (ROS) [30]. The physiological responses of seeds and seedling to CO treatment are reviewed in Figure 2.

## 6. CO—Crosstalk with Other Molecules

The CO signal transduction pathway functions through complex, non-linear crosstalk with other signaling molecules. A particularly notable interaction is observed between CO and NO. Their signaling pathways intersect in numerous cellular processes. These molecules are indispensable in the regulation of plants’ growth and development. As reported by Liu et al. [36], NO, functioning as a signaling molecule, assists in mitigating the deleterious effects of osmotic stress on wheat seed germination by interacting with the HO/CO signaling system. In a subsequent study, they reported that light can increase the activity of HO, *HO-1* gene expression, CO emission, and chlorophyll accumulation [37]. The increased activity is due to their sensitivity to zinc protoporphyrin (ZnPPIX) and HO-1 inhibitors. The regulation of the seedling HO/CO system by hematin and exogenous CO resulted in the de-etiolation of wheat seedlings. This effect was also achieved with the help of sodium nitroprusside (SNP), which is a NO donor. Furthermore, it has been demonstrated that the endogenous NO production in wheat seedling leaves is sensitive to light, hematin, and CO. These factors have been demonstrated to enhance NO production. The obtained results suggest the presence of a crosstalk between HO/CO and NO [37].

It has also been reported that CO acts as a signal to activate plant defense mechanisms against heat stress by stimulating NO production in wheat seedling roots. Treatment of *Triticum aestivum* seedlings with hemin resulted in a nearly twofold increase in NO levels and nitrate reductase (NR) activity in roots. The application of sodium tungstate, an inhibitor of NR, led to the counteraction of the increase in NO levels, thereby indicating the critical role of NR in NO production [46]. Conversely, agents that inhibit Ca^2+^ transfer from intracellular compartments to the cytoplasm, such as neomycin, have been observed to reduce NO levels and NR activity, suggesting a fundamental role of calcium ions in this signaling pathway [47]. The application of NO scavengers (PTIO) and NR inhibitors effectively prevented the activation of extracellular peroxidase and the accumulation of H_2_O_2_ in root tissue, thereby suggesting that NO contributes to the production of reactive oxygen species (ROS) [10]. Consequently, treatment with a CO donor reduced oxidative stress and increased the survival of wheat seedlings after exposure to high temperatures [46].

Additionally, evidence suggests that there is crosstalk between CO and NO during root growth in the presence of iron deficiency stress. The production of CO and NO in response to iron deficiency has been observed to induce alterations in cellular activities. Iron deficiency has been shown to increase heme oxygenase activity, leading to an elevated CO production. This increase in CO production subsequently leads to a change in the distribution of Pin-Formed 1 (PIN1) protein [48]. The function of PIN1 protein is to facilitate the transfer of auxin. It has been demonstrated that elevated auxin levels exert an influence on nitric oxide signaling, thereby augmenting root sensitivity to growth. This phenomenon has been shown to result in an augmentation of ferric chelate reductase (FCR) activity, a process that is imperative for the absorption of iron [49]. Furthermore, the presence of carbon monoxide (CO) and nitric oxide (NO) has been observed to enhance the expression of genes involved in iron absorption, including iron-regulated transporter1 (*IRT1*) and the ferric reduction oxidase 2 (*FRO2*) [50].

Furthermore, evidence has demonstrated that CO can promote the development of lateral roots in *Solanum lycopersicum* seedlings. This phenomenon is attributed to the activation of heme oxygenase 1 (LeHO-1), a process that leads to the production of intracellular CO. The increase in the protein level and the transcripts of this enzyme is associated with the CO increase in the lateral root. It was shown that CO is capable of stimulating the production of nitric oxide and auxin in root cells. Conversely, the effect of CO was found to be reduced by the NO scavenger cPTIO and the auxin transport inhibitor N-1-naphthylphthalamic acid. These findings suggest a collaborative, synergistic relationship between CO and NO in the regulation of lateral root growth [14].

The CO signal transduction pathway is not linear. As a signal molecule, CO exhibits extensive crosstalk with other signaling molecules, and its signal transmission pathway appears to be particularly closely connected to NO. These molecules play an important role in regulating plant growth and development, as well as their adaptation to adverse environmental conditions. It has been established that CO and NO interact in the light-induced de-etiolation process of plants. Light exposure has been demonstrated to enhance HO activity, leading to increased CO and NO production, and subsequently promote chlorophyll accumulation. This process has been found to be sensitive to zinc protoporphyrin (ZnPPIX), an inhibitor of HO-1, thereby underscoring a substantial crosstalk between the HO/CO system and NO [37].

The interplay between the HO/CO signaling system and NO has been observed during the process of wheat seed germination under condition of osmotic stress, resulting in a mitigation of damage [36]. This interplay has also been noted in the response to heat stress [46]. Subsequent research has demonstrated that CO functions as a secondary messenger, activating the nitrate reductase (NR) enzyme, thereby enhancing NO production. Consequently, the plants exhibited diminished sensitivity to heat stress [46].

It has also been reported that crosstalk between CO and NO promotes root growth under condition of iron deficiency stress [51]. Iron deficiency has been shown to induce increased HO activity and CO production, which in turn alters the distribution of Pin-Formed 1 (PIN1) protein [48], thereby affecting auxin transport. Elevated auxin levels have been shown to enhance nitric oxide signaling, thereby increasing root sensitivity and ferric chelate reductase (FCR) activity [49]. FCR is essential for iron absorption and the expression of genes involved in iron absorption, such as IRT1 and the ferric reduction oxidase 2 (*FRO2*) [50]. Furthermore, evidence suggests that crosstalk between CO and NO plays a crucial role in stimulating the growth of lateral roots in *Solanum lycopersicum* seedlings. In the context of lateral roots, elevated levels of LeHO-1 proteins and transcripts have been observed, resulting in augmented levels of CO and enhanced production of NO and auxin within root cells.

The formation of lateral roots is promoted by CO, which increases the levels of LeHO-1 proteins and transcripts. These proteins and transcripts are essential for CO production. This process is linked to auxin and NO, as an auxin transport inhibitor and a NO scavenger block CO’s effects. Additionally, CO treatment has been observed to increase overall IAA levels and enhance intracellular NO generation in tomato roots, suggesting a collaborative regulatory mechanism between CO and NO in modulating root architecture. The application of a NO scavenger, cPTIO, and an auxin transport inhibitor, N-1-naphthylphthalamic acid, has been observed to impede the effect of CO, further suggesting a synergistic relationship between CO and NO in the regulation of lateral root growth [14].

The regulatory mechanism of stomatal movement involves the interplay of CO/NO crosstalk and H_2_O_2_/CO interactions [52]. In *Vicia faba*, CO, akin to H_2_O_2_, prompts stomatal closure in a dose- and time-dependent manner. The findings suggest that CO-induced stomatal closure involves H_2_O_2_ signaling, as inhibitors of H_2_O_2_ production and scavengers of CO reverse the closure effect and reduce H_2_O_2_ fluorescence [52]. Furthermore, the analysis revealed that CO levels in guard cells are elevated under dark conditions, suggesting a role for CO in darkness-induced H_2_O_2_ synthesis. This process is facilitated by HO-1 and NADPH oxidase, which are pivotal enzymes in this pathway. However, NO has also been implicated in this process. The CO/NO scavenger hemoglobin (Hb) and the CO-specific inhibitor ZnPPIX, along with ascorbic acid (ASA) and diphenylene iodonium (DPI), reverse the darkness-induced stomatal closure and H_2_O_2_ fluorescence. This finding suggests that NO, akin to CO, contributes to the signaling pathway that regulates stomatal movement, particularly under dark conditions [52].

The CO signaling pathway has also been observed to interact with hydrogen sulfide (H_2_S) signaling [53]. Li and Gu [54] demonstrated that CO pretreatment significantly enhances the heat stress tolerance of tobacco cell suspensions by increasing endogenous H_2_S levels. Their findings suggest that CO boosts H_2_S production indirectly by activating L-cysteine desulfhydrase, the enzyme responsible for H_2_S synthesis. Consequently, H_2_S has been demonstrated to enhance stress tolerance by activating plant defense mechanisms.

In a recent study, Jin et al. [23] reported that H_2_ can act as an inducer of HO. Furthermore, they demonstrated that seed pretreatment of *Medicago sativa* seeds with H_2_ resulted in increased HO-1 transcript levels and heightened heme oxygenase enzyme activity under paraquat-induced oxidative stress. The addition of CO solution led to an increase in the effects of H_2_, and the addition of heme oxygenase inhibitors led to a decrease in the effects of H_2_.

## 7. Conclusions

There is ample evidence to support the notion that CO, along with other signaling molecules, plays a substantial role in regulating plant growth and development. This molecule has been demonstrated to stimulate the germination of seeds and alleviate their dormancy. Furthermore, CO enhances the ability of seeds and seedlings to withstand environmental stresses by improving defense mechanisms, such as boosting antioxidant activity, photosynthetic efficiency, cell membrane stability, and leaf water status. The multifaceted effects of CO on seed physiology and biochemistry are summarized in Figure 2.

Furthermore, CO has been observed to interact with other plant signaling molecules, thereby contributing to the reinforcement of defense responses. However, the molecular mechanisms underlying CO’s action in plants remain incompletely understood, as does its precise role in plant biological processes. To address these gaps, further studies are necessary, particularly focusing on gene expression regulation under exogenously applied CO. Investigations using mutant lines with silenced or enhanced expression of genes involved in CO biosynthesis and degradation could provide valuable insights.

Another challenge lies in identifying appropriate chemical probes to visualize the presence and localization of CO within biological systems using microscopy. Comprehensive research employing diverse experimental approaches is essential to elucidate these unanswered questions and advance our understanding of CO’s role in plant biology.

## References

[1] Feng L., Wei L., Liu Y., Ren J., Liao W. (2023). Carbon monoxide/heme oxygenase system in plant: Roles in abiotic stress response and crosstalk with other signals molecules. Nitric Oxide.

[2] Hopper C.P., Zambrana P.N., Goebel U., Wollborn J. (2021). A brief history of carbon monoxide and its therapeutic origins. Nitric Oxide.

[3] Richards H.M., MacDougal D.T. (1904). The influence of carbon monoxide and other gases upon plants. Bull. Torrey Bot. Club.

[4] He D., Deng G., Ying S., Yang W., Wei J., Li P. (2020). Carbon monoxide signal breaks primary seed dormancy by transcriptional silence of DOG1 in *Arabidopsis thaliana*. Phyton.

[5] Wang M., Liao W. (2016). Carbon monoxide as a signaling molecule in plants. Front. Plant Sci..

[6] Wu M.Z., Huang J.J., Xu S., Ling T.F., Xie Y.J., Shen W.B. (2010). Heme oxygenase delays programmed cell death in wheat aleurone layers by modulation of hydrogen peroxide metabolism. J. Exp. Bot..

[7] Xie Y.J., Zhang C., Lai D.W., Sun Y., Samma M.K., Zhang J., Chen W. (2014). Hydrogen sulfide delays GA-triggered programmed cell death in wheat aleurone layers by the modulation of glutathione homeostasis and heme oxygenase-1 expression. J. Plant Physiol..

[8] Wojtyla Ł., Lechowska K., Kubala S., Garnczarska M. (2016). Different modes of hydrogen peroxide action during seed germination. Front. Plant Sci..

[9] Wei Y.Y., Zheng Q., Liu Z.P., Yang Z.M. (2011). Regulation of tolerance of Chlamydomonas reinhardtii to heavy metal toxicity by heme oxygenase-1 and carbon monoxide. Plant Cell Physiol..

[10] Kolupaev Y.E., Shkliarevskyi M.A., Karpets Y.V., Shvidenko N.V., Lugovaya A.A. (2021). ROS-dependent induction of antioxidant system and heat resistance of wheat seedlings by hemin, Russ. J. Plant Physiol..

[11] Dey P., Pattanaik D., Mohapatra D., Saha D., Dash D., Mishra A., Priyadarshinee L., Singh A., Swain P., Baig M. (2024). Gasotransmitters signaling and their crosstalk with other signaling molecules under diverse stress conditions in plants. S. Afr. J. Bot..

[12] Gisk B., Yasui Y., Kohchi T., Frankenberg-Dinkel N. (2010). Characterization of the heme oxygenase protein family in *Arabidopsis thaliana* reveals a diversity of functions. Biochem. J..

[13] Mathur P., Subba R., Mukherjee S. (2023). Carbon Monoxide (CO) and Its Association with Other Gasotransmitters in Root Development, Growth and Signaling. Plant in Challenging Environments.

[14] Guo K., Xia K., Yang Z.M. (2008). Regulation of tomato lateral root development by carbon monoxide and involvement in auxin and nitric oxide. J. Exp. Bot..

[15] Guo K., Kong W.W., Yang Z.M. (2009). Carbon monoxide promotes root hair development in tomato. Plant Cell Environ..

[16] Xu J., Xuan W., Huang B., Zhou Y., Ling T., Xu S., Shen W. (2006). Carbon monoxide-induced adventitious rooting of hypocotyl cuttings from mung bean seedling. Chin. Sci. Bull..

[17] Ikebudu V.C., Nkuna M., Ndou N., Ajayi R.F., Chivasa S., Cornish K., Mulaudzi T. (2024). Carbon Monoxide Alleviates Salt-Induced Oxidative Damage in *Sorghum bicolor* by Inducing the Expression of Proline Biosynthesis and Antioxidant Genes. Plants.

[18] Liu K., Xu S., Xuan W., Ling T., Cao Z., Huang B., Sun Y., Fang L., Liu Z., Zhao N. (2007). Carbon monoxide counteracts the inhibition of seed germination and alleviates oxidative damage caused by salt stress in *Oryza sativa*. Plant Sci..

[19] Xie Y., Ling T., Han Y.I., Liu K., Zheng Q., Huang L., Yuan X., He Z., Hu B., Fang L. (2008). Carbon monoxide enhances salt tolerance by nitric oxide-mediated maintenance of ion homeostasis and up-regulation of antioxidant defence in wheat seedling roots. Plant Cell Environ..

[20] Anita M.N., Shekhawat G.S. (2024). Hemin in Plants: Biosynthesis and Role in ROS Detoxification During Oxidative Stress. Plant Growth Regulators, Resilience for Sustainable Agriculture.

[21] Liu Y., Xu J., Lu X., Huang M., Mao Y., Li C., Yu W., Li C. (2024). Carbon monoxide is involved in melatonin-enhanced drought resistance in tomato seedlings by enhancing chlorophyll synthesis pathway. BMC Plant Biol..

[22] Chen J., Yang Z.M. (2011). Enhancement of tolerance of Indian mustard (*Brassica juncea*) to mercury by carbon monoxide. J. Hazard. Mater..

[23] Jin Q., Zhu K., Cui W., Xie Y., Han B.I.N., Shen W. (2013). Hydrogen gas acts as a novel bioactive molecule in enhancing plant tolerance to paraquat-induced oxidative stress via the modulation of heme oxygenase-1 signalling system. Plant Cell Environ..

[24] Huang B.K., Xu S., Xuan W., Li M., Cao Z.Y., Liu K.L., Ling T.F., Shen W.B. (2006). Carbon monoxide alleviates salt-induced oxidative damage in wheat seedling leaves. J. Integr. Plant Biol..

[25] Ling T., Zhang B., Cui W., Wu M., Lin J., Zhou W., Huang J., Shen W. (2009). Carbon monoxide mitigates salt-induced inhibition of root growth and suppresses programmed cell death in wheat primary roots by inhibiting superoxide anion overproduction. Plant Sci..

[26] Yun F., Huang D., Zhang M., Wang C., Deng Y., Gao R., Hou X., Liu Z., Liao W. (2022). Comprehensive transcriptome analysis unravels the crucial genes during adventitious root development induced by carbon monoxide in *Cucumis sativus* L.. Mol. Biol. Rep..

[27] Weng X., Zhu L., Yu S., Liu Y., Ru Y., Zhang Z., He Z., Zhou L., Chen X. (2022). Carbon monoxide promotes stomatal initiation by regulating the expression of two EPF genes in Arabidopsis cotyledons. Front. Plant Sci..

[28] Jia Y., Li R., Yang W., Chen Z., Hu X. (2018). Carbon monoxide signal regulates light-initiated seed germination by suppressing SOM expression. Plant Sci..

[29] Kong W.W., Zhang L.P., Guo K., Liu Z.P., Yang Z.M. (2010). Carbon monoxide improves adaptation of Arabidopsis to iron deficiency. Plant Biotechnol. J..

[30] Bai X.G., Chen J.H., Kong X.X., Todd C.D., Yang Y.P., Hu X.Y., Li D.Z. (2012). Carbon monoxide enhances the chilling tolerance of recalcitrant *Baccaurea ramiflora* seeds via nitric oxide-mediated glutathione homeostasis. Free Radic. Biol. Med..

[31] Zhang C., Li Y., Yuan F., Hu S., He P. (2012). Effects of hematin and carbon monoxide on the salinity stress responses of *Cassia obtusifolia* L. seeds and seedlings. Plant Soil.

[32] Chen Y., Wang M., Hu L., Liao W., Dawuda M.M., Li C. (2017). Carbon monoxide is involved in hydrogen gas-induced adventitious root development in cucumber under simulated drought stress. Front. Plant Sci..

[33] Xuan W., Zhu F.Y., Xu S., Huang B.K., Ling T.F., Qi J.Y., Ye M.B., Shen W.B. (2008). The heme oxygenase/carbon monoxide system is involved in the auxin-induced cucumber adventitious rooting process. Plant Physiol..

[34] Cheng T., Hu L., Wang P., Yang X., Peng Y., Lu Y., Chen J., Shi J. (2018). Carbon monoxide potentiates high temperature-induced nicotine biosynthesis in tobacco. Int. J. Mol. Sci..

[35] Dekker J., Hargrove M. (2002). Weedy adaptation in Setaria spp. V. Effects of gaseous environment on giant foxtail (*Setaria faberii*) (Poaceae) seed germination. Am. J. Bot..

[36] Liu Y., Xu S., Ling T., Xu L., Shen W. (2010). Heme oxygenase/carbon monoxide system participates in regulating wheat seed germination under osmotic stress involving the nitric oxide pathway. J. Plant Physiol..

[37] Liu Y., Li X., Xu L., Shen W. (2013). De-etiolation of wheat seedling leaves: Cross talk between heme oxygenase/carbon monoxide and nitric oxide. PLoS ONE.

[38] Sa Z.S., Huang L.Q., Wu G.L., Ding J.P., Chen X.Y., Yu T., Shi C., Shen W.B. (2007). Carbon monoxide: A novel antioxidant against oxidative stress in wheat seedling leaves. J. Integr. Plant Biol..

[39] Cao Z., Huang B., Wang Q., Xuan W., Ling T., Zhang B., Chen X., Li N., Shen W. (2007). Involvement of carbon monoxide produced by heme oxygenase in ABA-induced stomatal closure in *Vicia faba* and its proposed signal transduction pathway. Chin. Sci. Bull..

[40] Song X.G., She X.P., Zhang B. (2008). Carbon monoxide-induced stomatal closure in *Vicia faba* is dependent on nitric oxide synthesis. Physiol. Plant..

[41] Li X., Chen T., Li Y., Wang Z., Cao H., Chen F., Li Y., Soppe W.J.J., Li W., Liu Y. (2019). ETR1/RDO3 regulates seed dormancy by relieving the inhibitory effect of the ERF12-TPL complex on DELAY OF GERMINATION1 expression. Plant Cell.

[42] Fu Y., Ma L., Li J., Hou D., Zeng B., Zhang L., Liu C., Bi Q., Tam J., Yu X. (2024). Factors Influencing Seed Dormancy and Germination and Advances in Seed Priming Technology. Plants.

[43] Nakabayashi K., Bartsch M., Xiang Y., Miatton E., Pellengahr S., Yano R., Seo M., Soppe W.J.J. (2012). The time required for dormancyrelease in *Arabidopsis* is determined by delay of germination1 protein levels in freshly harvested seeds. Plant Cell.

[44] Lim S., Park J., Lee N., Jeong J., Toh S., Watanabe A., Kim J., Kang H., Kim D.H., Kawakami N. (2013). ABA-insensitive3, ABA-insensitive5, and DELLAs Interact to activate the expression of SOMNUS and other high-temperature-inducible genes in imbibed seeds in *Arabidopsis*. Plant Cell.

[45] Amooaghaie R., Tabatabaie F. (2017). Osmopriming-induced salt tolerance during seed germination of alfalfa most likely mediates through H_2_O_2_ signaling and upregulation of heme oxygenase. Protoplasma.

[46] Shkliarevskyi M.A., Kolupaev Y.E., Karpets Y.V., Lugovaya A.A., Bessonova V.P. (2021). Involvement of nitrate reductase and nitric oxide (NO) in implementation of the stress-protective action of a carbon monoxide (CO) donor on wheat seedlings under hyperthermy. Russ. J. Plant Physiol..

[47] Shkliarevskyi M.A., Karpets Y.V., Kolupaev Y.E., Lugovaya A.A., Dmitriev A.P. (2020). Calcium-dependent changes in cellular redox homeostasis and heat resistance of wheat plantlets under the influence of hemin (carbon monoxide donor). Cytol. Genet..

[48] Li J., Zhang Q., Chen H., Xu D., Chen Z., Wen Y. (2022). Role of heme oxygenase-1 in dual stress response of herbicide and micronutrient Fe in *Arabidopsis thaliana*. J. Agric. Food Chem..

[49] Buet A., Simontacchi M. (2015). Nitric oxide and plant iron homeostasis. Ann. N. Y. Acad. Sci..

[50] Hong K., Radani Y., Ahmad W., Li P., Luo Y. (2024). Carbon Monoxide Modulates Auxin Transport and Nitric Oxide Signaling in Plants under Iron Deficiency Stress. Phyton.

[51] Singh N., Bhatla S.C. (2022). Heme oxygenase-nitric oxide crosstalk-mediated iron homeostasis in plants under oxidative stress. Free Radic. Biol. Med..

[52] She X.P., Song X.G. (2008). Carbon monoxide-induced stomatal closure involves generation of hydrogen peroxide in *Vicia faba* guard cells. J. Integr. Plant Biol..

[53] Shen J., Zhang J., Zhou M.J., Zhou H., Cui B.M., Gotor C., Xie Y.J. (2020). Persulfdation-based modifcation of cysteine desulfhydrase and the NADPH oxidase RBOHD controls guard cell abscise acid signaling. Plant Cell.

[54] Li Z.G., Gu S.P. (2016). Hydrogen sulfide as a signal molecule in hematin-induced heat tolerance of tobacco cell suspension. Biol. Plant..

